# Retraction: An Eocene army ant

**DOI:** 10.1098/rsbl.2023.0059

**Published:** 2023-03-22

**Authors:** Christine E. Sosiak, Marek L. Borowiec, Phillip Barden

**Affiliations:** ^1^ Federated Department of Biological Sciences, New Jersey Institute of Technology, Newark, NJ 07102-1982, USA; ^2^ Department of Agricultural Biology and C. P. Gillette Museum of Arthropod Diversity, Colorado State University, Fort Collins, CO, USA; ^3^ Division of Invertebrate Zoology, American Museum of Natural History, New York, NY, USA

Shortly after the publication of this report the authors were contacted independently by two researchers (Brendon E. Boudinot, The University of Jena and Dmitry A. Dubovikoff, St Petersburg State University). Both researchers indicated that they were aware of several specimens of *Dorylus* preserved in sub-fossil resin (copal), within historical collections of Baltic amber from 1920 to 1930s—the same time frame associated with the Museum of Comparative Zoology (MCZ) specimen reported in Sosiak *et al.* [1]. Copal in both cases were either initially misidentified or mixed among pieces of authentic Baltic amber. The collection at the University of Jena comprised both Baltic amber and copal mixed and without labels; those copal specimens were identified via FTIR analysis this year. At least one of the Jena copal specimens, although not a *Dorylus* army ant specimen, was prepared in the same manner as the MCZ specimen we reported here: trimmed and mounted directly to a microscope slide. The traditional practice of slide mounting Baltic amber with inclusions contributed to our confidence regarding the provenance of the MCZ specimen. Slide mounting was a common practice in the nineteenth and early twentieth century among Baltic amber researchers [2].

To assess the identity of the MCZ specimen, we compiled a reference series of definitive Baltic amber (*n* = 2), Tanzanian copal (*n* = 2) and Zanzibar copal (*n* = 2) from the American Museum of Natural History. Baltic amber specimens comprised both crude amber collected directly from the Palmnicken mine as well as a historic prepared specimen with an index inclusion, the ant *Ctenobethylus goeppperti*, which is common in Baltic amber [3]. Sample spectra were characterized using an Agilent Cary 620 FTIR Microscope at the Center for Environmental Engineering and Science at the New Jersey Institute of Technology. Samples were prepared and analysed by an FTIR specialist previously unaffiliated with the study, and specimens were provided to the specialist without any identifying labels. Between two and four samples were analysed for each reference specimen as well as the MCZ specimen.

While we recovered a characteristic signature of Baltic amber in all spectra from both raw and prepared Baltic amber references (the ‘Baltic shoulder' between 1175 and 1250 cm^−1^ [4]), we did not recover the ‘shoulder' in the MCZ specimen or in any of the copal specimens (figure 1). The exact provenance of this specimen will require additional investigation; however, we feel confident in stating that the MCZ holotype is not Baltic amber and is likely a sub-fossil resin. Because the age and locality of Baltic amber is central to the conclusions made in our report, we wish to retract our publication.

**Figure 1.**
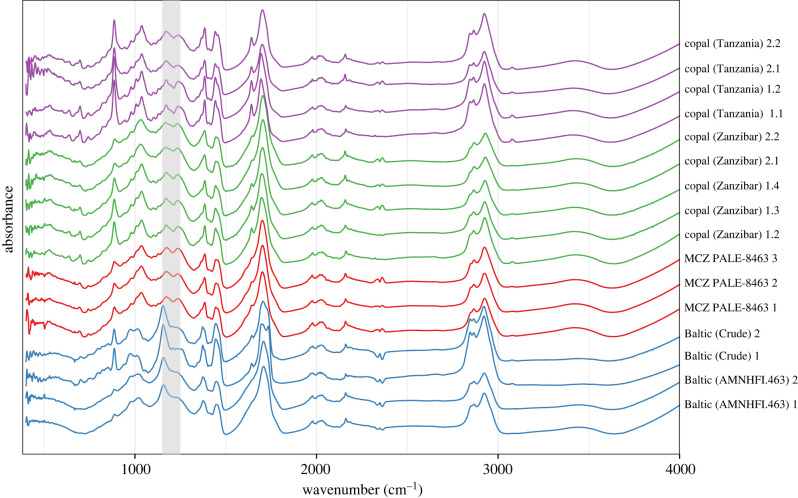
Comparative FTIR spectra of reference and MCZ PALE-8463 holotype. ATR spectra were baseline corrected using the ‘modployfit' method and normalized by total intensity in the R package ChemoSpec [5]. Colours denote specimen source; blue: Baltic amber, red: MCZ PALE-8463 holotype, green: Zanzibar copal, purple: Tanzania copal. Numbers indicate specimen number where appropriate followed by within-specimen sample number. Grey-shaded region corresponds to 1150–1250 cm^−1^, which encompasses the Baltic shoulder [5].

Our report includes taxonomic activity that, under the International Code of Zoological Nomenclature, will not be invalidated by retraction [6]. In the light of the much more recent age of the specimen, synonymy of *Dissimulodorylus* with *Dorylus* appears appropriate. We have compared the type specimen of *Dissimulodorylus perseus* to extant *Dorylus* species, including specimens reported from copal, and have not identified a clear justification for species synonymy based on current data. It may be the case that this putative subfossil material has preserved an instance of recent extinction, as has been documented in some copal bees from the region [7], or simply an extant species as yet unfound, however, additional data are needed.

It is unclear to what extent copal from the early twentieth century was disseminated to collections as Baltic amber. The presence of this material across three museums and three countries, and several similar cases in UK museums [8] is cause for urging scrutiny and caution with historical collections of Baltic amber.

Our initial confidence in the provenance of this specimen was based primarily on museum labels and records as well as our perceived lack of morphological affinities between this taxon and any extant *Dorylus*. Had we been aware of *Dorylus* sub-fossil material erroneously mixed with Baltic amber in other historic collections, we would have undertaken FTIR analyses early in our work, however such analysis is not routinely incorporated into amber species descriptions. Across the approximately 130 described Baltic amber ant species, we are not aware of any instances of FTIR data accompanying species descriptions, including instances where Baltic amber species revealed disjunct distributions or local extinction. While museum labels remain the most common manner in which specimen provenance is established, we should have exhibited more caution regarding specimen provenance and deeply regret this error.

## Data Availability

Raw FTIR data are available from the Dryad Digital Repository: https://doi.org/10.5061/dryad.rn8pk0pgt [9].
